# Publisher Correction: Implicit motor learning within three trials

**DOI:** 10.1038/s41598-021-88889-y

**Published:** 2021-04-21

**Authors:** Jennifer E. Ruttle, Bernard Marius ’t Hart, Denise Y. P. Henriques

**Affiliations:** 1 Centre for Vision Research, York University, Toronto, Canada; 2 Department of Psychology, York University, Toronto, Canada; 3 School of Kinesiology and Health Science, York University, Toronto, Canada

Correction to: *Scientific Reports* 10.1038/s41598-021-81031-y, published online 15 January 2021

In the original version of this Article, the author Bernard Marius ’t Hart was incorrectly indexed. This error has now been corrected.

